# Enamel Ultrastructure in Fossil Cetaceans (Cetacea: Archaeoceti and Odontoceti)

**DOI:** 10.1371/journal.pone.0116557

**Published:** 2015-01-28

**Authors:** Carolina Loch, Jules A. Kieser, R. Ewan Fordyce

**Affiliations:** 1 Department of Geology, University of Otago, Dunedin, New Zealand; 2 Sir John Walsh Research Institute, Faculty of Dentistry, University of Otago, Dunedin, New Zealand; NYIT College of Osteopathic Medicine, UNITED STATES

## Abstract

The transition from terrestrial ancestry to a fully pelagic life profoundly altered the body systems of cetaceans, with extreme morphological changes in the skull and feeding apparatus. The Oligocene Epoch was a crucial time in the evolution of cetaceans when the ancestors of modern whales and dolphins (Neoceti) underwent major diversification, but details of dental structure and evolution are poorly known for the archaeocete-neocete transition. We report the morphology of teeth and ultrastructure of enamel in archaeocetes, and fossil platanistoids and delphinoids, ranging from late Oligocene (Waitaki Valley, New Zealand) to Pliocene (Caldera, Chile). Teeth were embedded in epoxy resin, sectioned in cross and longitudinal planes, polished, etched, and coated with gold palladium for scanning electron microscopy (SEM) observation. SEM images showed that in archaeocetes, squalodontids and Prosqualodon (taxa with heterodont and nonpolydont/limited polydont teeth), the inner enamel was organized in Hunter-Schreger bands (HSB) with an outer layer of radial enamel. This is a common pattern in most large-bodied mammals and it is regarded as a biomechanical adaptation related to food processing and crack resistance. Fossil Otekaikea sp. and delphinoids, which were polydont and homodont, showed a simpler structure, with inner radial and outer prismless enamel. Radial enamel is regarded as more wear-resistant and has been retained in several mammalian taxa in which opposing tooth surfaces slide over each other. These observations suggest that the transition from a heterodont and nonpolydont/limited polydont dentition in archaeocetes and early odontocetes, to homodont and polydont teeth in crownward odontocetes, was also linked to a marked simplification in the enamel Schmelzmuster. These patterns probably reflect functional shifts in food processing from shear-and-mastication in archaeocetes and early odontocetes, to pierce-and-grasp occlusion in crownward odontocetes, with the implication of less demanding feeding biomechanics as seen in most extant odontocetes.

## Introduction

Cetaceans—whales, dolphins—are unusual mammals that are adapted to a fully aquatic life through a range of physiological and morphological modifications in respiration, circulation, feeding and locomotion [1]. The evolutionary history of the group began with the rise of archaeocetes (archaic stem Cetacea) in the ancient Tethys seaway in the Early Eocene more than 50 million years ago [2]. The earliest fossils include amphibious species from Eocene sediments of fluvial, brackish and fully marine origin. Crown cetaceans (Odontoceti and Mysticeti, or Neoceti) appeared probably in the late Eocene, by 35–36 Ma, and diversified rapidly during the Oligocene. Odontocetes and mysticetes most likely originated from a group of widespread pelagic archaeocetes, the Basilosauridae [3,4].

Archaeocetes typically had elongated rostra bearing a heterodont dentition. The anterior teeth were conical, high-crowned, and separated by prominent diastemata, while the posterior teeth were lower-crowned, more-heavily built and, in the later-appearing group Basilosauridae, ornamented with multiple neomorphic denticles on the mesial and distal keels. Archaeocetes were diphyodont, with no evidence for the polydonty seen in crown Cetacea [5]. The morphology of their teeth and feeding apparatus suggest that most archaeocetes could shear and also snap prey items [3,6].

Crown Cetacea (Mysticeti and Odontoceti) are polydont and monophyodont. Extant mysticetes, however, have their set of multiple tooth buds resorbed before birth and bear filtering baleen in their upper jaws from later foetal stage [7]. Most Odontoceti (fossil and living) have elongated rostra bearing a polydont dentition. Archaic odontocetes were heterodont and polydont, with an accompanying trend towards homodonty and increased polydonty during the evolution of the group. In contrast, some fossil and extant odontocetes developed short, broad and robust rostra (*e.g.* extant *Globicephala* and extinct *Prosqualodon*), and reduced both the number of teeth and the role of teeth in food processing (*e.g.* in the groups Ziphiidae, Kogiidae, Monodontidae, Globicephalinae) [3,8]. For most living odontocetes, the feeding apparatus is specialized for food acquisition, with little or no reduction and processing of food in the oral cavity. Many odontocete species grasp and eat prey whole, but some species may slash or tear pieces from large prey; many species may use suction feeding to catch and transport food items [9]. The morphology of teeth and wear patterns suggest that archaic heterodont odontocetes used their teeth for more than just grasping and eating whole prey; teeth often functioned for the mechanical reduction of food items taken and for predation on hard food [3].

Teeth form a prominent part of mammal remains in paleontological and archaeological sites because of the good preservation potential conferred by high mineral content and density of enamel and dentine compared to other skeletal elements. Further, teeth have long been used to elucidate aspects of the ecology, functional morphology and systematics of fossil and recent mammal species [10]. Teeth play a major role in studies of dietary adaptations, and may elucidate behaviors including social activities, defense and sexual signaling, representing a powerful tool in mammalian evolutionary biology [11,12].

Enamel is a highly mineralized tissue that forms the outermost layer of reptilian and mammalian tooth crowns. Mammalian enamel is organized into bundles of bounded hydroxyapatite crystals known as enamel prisms, which can vary in morphology, diameter, density, patterns of organization, and packaging [13]. The 3-dimensional arrangement of enamel types may vary between different taxa or different tooth types in heterodont species [13]. The diversity of complex structures in mammalian enamel reflects many determinants, particularly biomechanical/functional adaptation, geometric packing, and phylogeny. The relationship between biomechanical stresses and enamel structures reflects functional constraints; however phylogeny may influence enamel differentiation and variability, as some specific structures are closely correlated with particular mammalian taxa [14]. Many previous studies have sought to describe the enamel ultrastructure in fossil mammals to address issues of systematics and functional morphology/lifestyle (*e.g.* [11,15–17]).

Structural changes in cetacean teeth occurred during adaptation of the skeletal and body systems to an increasingly pelagic lifestyle, as considered in other studies on cetacean teeth. The ultrastructure of enamel has been studied for Eocene basal archaeocetes [18–20], while the enamel of recent odontocetes was reportedly both prismatic and prismless [20–22]. Details are virtually unknown for cetacean dental structure and evolution in Oligocene times, when there was a major diversification of the Neoceti (Mysticeti and Odontoceti). The early echolocating dolphins and filter feeding whales rapidly diversified in response to changing ocean ecosystems and new ecological opportunities. A few relict Oligocene archaeocetes are also known. Modern families of mysticetes and odontocetes appeared progressively from possibly the late Oligocene, and definitely the early Miocene to the Pliocene [3,4].

In spite of the ever-increasing diversity of fossil species, there are few published accounts of dental and enamel ultrastructure in fossil Neoceti. A major limitation in such work is the need for destructive sampling. Well-preserved fossil cetacean skulls with teeth in place are uncommon, scientifically valuable, and are not usually available from Museums for destructive sampling, as the literature on tooth structure reveals. Skulls are conserved because they are fundamental to identify the species, and to use in studies of phylogeny and function. Isolated teeth, conversely, are relatively common and more-readily available for destructive sectioning, but unless associated with a taxonomically-diagnostic skull, the exact species is commonly uncertain. Nevertheless, the genus or family can often be identified based on the similarity of an isolated tooth to teeth seen in situ in identifiable skulls, especially for more-archaic Cetacea which have feature-laden heterodont teeth, and especially for localities or strata which have already produced diverse assemblages of reliably identified toothed cetaceans.

This study describes the arrangement and ultrastructure of teeth in 7 species of fossil cetaceans of Oligocene to Pliocene age. Our main focus is on enamel, with brief accounts on the structure of dentine and on the diagenesis of tooth tissues. Functional aspects and biomechanical implications of the enamel arrangement in fossil cetaceans are examined and discussed, with reference to extant cetaceans and other mammals.

## Material and Methods

### Material Examined

Fossil cetacean teeth analyzed here are from archaeocetes, platanistoids and delphinoids, and were collected in strata ranging in age from late Oligocene (Waitaki Valley, New Zealand) to Pliocene (Caldera, Chile). Specimens were made available for destructive sampling from the collections of the Geology Museum, University of Otago (Dunedin, New Zealand—OU), Museo Nacional de Historia Natural de Chile (Santiago, Chile—SGO-PV) and Museo Paleontológico Egidio Feruglio (Trelew, Argentina—MPEF-PV). One specimen belongs to the formally named species, *Prosqualodon australis*. For the other specimens not named to species-level (Table 1), the teeth of OU 22023, OU 22457, OU 22257, OU 22306, OU 22108 and SGO-PV-754 were associated with taxonomically-diagnostic rostra or skulls and/or tympanoperiotics. A list of key features which diagnose the study teeth to family-level is in the Supporting Information (S1 Table). No permits were required for the described study, which complied with all relevant regulations.

**Table 1:** Fossil cetaceans analyzed.

**Species**	**Collection number**	**Tooth type**	**Provenance and age**
**Archaeoceti**			
unnamed Kekenodontidae	OU 22023	Molar crown, denticulate	Kokoamu Greensand, Waihao, NZ; late Oligocene
**Platanistoidea**			
unnamed Squalodontidae	OU 22457	Incisor or canine crown, single-cusped	Otekaike Limestone, Milburn, NZ; late Oligocene
unnamed Squalodontidae	OU 22257	Cheek tooth, denticulate	Otekaike Limestone, Hakataramea, NZ; late Oligocene
unnamed species of *Otekaikea*	OU 22306	Tooth crown and root, position uncertain, single-cusped	Otekaike Limestone, Hakataramea, NZ; Oligocene-Miocene boundary
*Prosqualodon australis*	MPEF-PV 1868	Cheek tooth, denticulate	Gaiman Formation, Argentina; early Miocene
**Delphinoidea**			
unnamed Delphinoidea	OU 22108	Tooth crown, position uncertain, single-cusped	lower Otekaike Limestone, Hakataramea, NZ; late Oligocene
unnamed Delphinoidea	SGO-PV-754	Tooth crown, position uncertain, single cusped	Bahía Inglesa Formation, Chile; early Pliocene

## Methods

**Figure 1:**
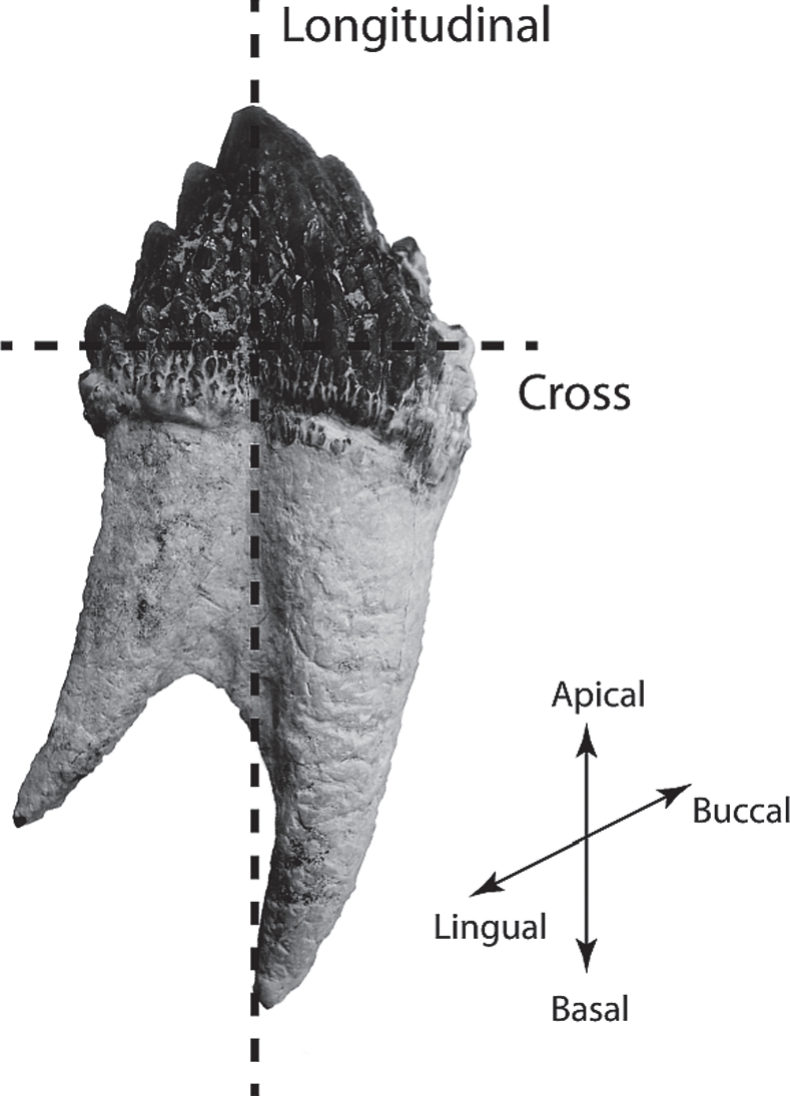
Cross and longitudinal sectioning planes in fossil cetacean teeth.

Silicone rubber molds of the study teeth were made, and epoxy resin replicas produced, to record the original 3-dimensional structure before destructive sampling; replicas are held in the Geology Museum, University of Otago. Fossil cetacean teeth were surface-cleaned with alcohol and embedded in epoxy resin (Epofix Cold-Setting Embedding Resin, Struers, Copenhagen, Denmark) using silicon molds. After setting for 24 hours, specimens were sectioned using a MOD13 diamond wheel in an Accutom-50 high-speed saw (Struers, Copenhagen, Denmark) under water irrigation. Cross-sectional and longitudinal sections (Fig. 1) were polished in a TegraPol-21 polisher (Struers, Copenhagen, Denmark) with silicon carbide paper (1200, 2400 and 4000 grit) and sonicated in water in an ultrasonic cleaner for three minutes after each of the polishing sessions. Final polishing was achieved with 3 μm and 1 μm diamond paste. Specimens were then sonicated in ethanol for one minute for final removal of any residual debris.

To reveal detailed enamel structure, fossil dental specimens were etched with 2M hydrochloric acid for up to 10 seconds following Von Koenigswald et al. [17], and sonicated in water for one minute, to avoid damage to fragile specimens. Samples were then coated with gold palladium for scanning electron microscopy (SEM) observation. Secondary and backscatter electron microscopic images were obtained in a JEOL JSM-6700F Field Emission SEM (JEOL Ltd., Tokyo, Japan), operating at 5 kV and 10 μA. Magnifications in the SEM ranged from 30X to 5000X.

### Sampling practicalities

Previous studies of enamel ultrastructure in mammals have demonstrated that overall enamel organization is generally consistent among individuals of the same species, but may vary among species [15,19]. All taxa in this study were represented by a single specimen because of the rarity of fossils readily identifiable to taxon and also available for destructive sampling. We presume that the structural features described were typical of each species, but the conclusions were made with some caution. Here we mainly analyzed the shape and size of prisms and prism sheaths, the spatial organization of prisms and interprismatic matrix, and the overall organization of the enamel in each tooth (according to Carlson and Krause, Maas and Thewissen [15,19]). Anatomical terminology followed Von Koenigswald and Sander [23].

## Results

### Macromorphology

**Figure 2:**
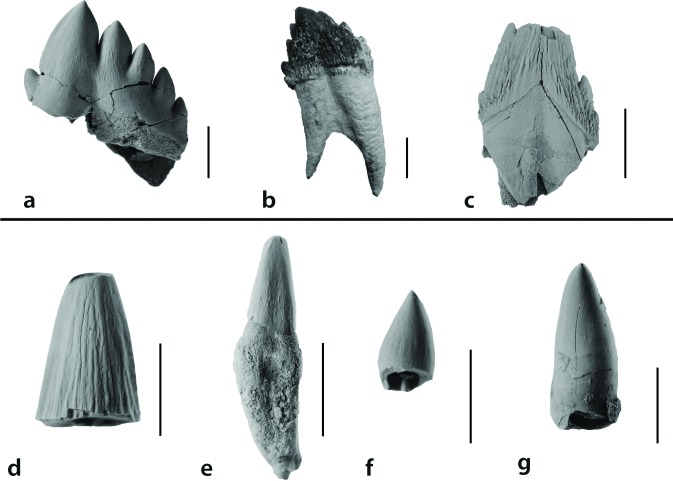
Specimens examined in lingual view. All fossils other than *Prosqualodon australis* have been coated with sublimed ammonium chloride. a) Unnamed Kekenodontidae (OU 22023). b) *Prosqualodon australis* (MPEF-PV 1868). c) Unnamed Squalodontidae (OU 22257). d) Unnamed Squalodontidae (OU 22457). e) *Otekaikea* sp. (cf. Squalodelphinidae) (OU 22306). f) Unnamed Delphinoidea (OU 22108). g) Unnamed Delphinoidea (SGO-PV-754). Scale bars = 1 cm. Images of Fig. 2A and 2C–F used with permission of Geology Museum, University of Otago.

Teeth analyzed in this study consisted of diverse tooth types. Cheek teeth (premolar and molar equivalents) were analyzed for the Waihao Kekenodontidae (OU 22023), the Hakataramea Squalodontidae (OU 22257) and the Patagonian *Prosqualodon australis* (Fig. 2A–C). These teeth had a main cusp and small accessory denticles on the anterior (mesial) and posterior (distal) faces. The anterior face had fewer denticles than the posterior. The crown was bucco-lingually compressed and in the OU 22257 Squalodontidae and *Prosqualodon* it was ornamented with *cristae rugosae* (*sensu* Rothausen [24]), which were more prominent at the base of the crown, where enamel nodules were formed. The other Squalodontidae specimen examined (Milburn tooth; OU 22457), was represented by an incisor crown which was triangular and subconical, bucco-lingually compressed, with vertical subparallel ridges on the enamel, and without accessory denticles (Fig. 2D).

Less complex tooth morphologies were observed in *Otekaikea* sp. (cf. Squalodelphinidae) and Delphinoidea. *Otekaikea* sp. (OU 22306) had a slender simple crown, conical in shape, bucco-lingually compressed and gently curved lingually (Fig. 2E). The two Delphinoidea analyzed (Hakataramea tooth—OU 22108; and SGO-PV-754) had a conical and featureless dental crown also curved lingually (Fig. 2F and G). The external surface of the enamel was featureless at macroscopic scale in both *Otekaikea* sp. and the Delphinoidea specimens.

### Enamel thickness and overall organization

Enamel thickness varied among the species analyzed (Table 2). The thinnest enamel was observed in the conical-crowned *Otekaikea* sp. OU 22306 and in the Delphinoidea OU 22108. Moderately thick enamel was observed both in the denticulate Kekenodontidae OU 22023 and in the geologically young conical-crowned Chilean Delphinoidea SGO-PV-754. Thicker enamel was found in the squalodontids OU 22257 (denticulate crown, cheek-tooth) and OU 22457 (conical crown, incisor), as well as in the denticulate *Prosqualodon australis.* On average, enamel thickness varied from 350–380 μm in these specimens, but it reached up to 580 μm in thickness in zones where enamel was heavily wrinkled to form strong subvertical ridges.

**Table 2:** Enamel thickness in the fossil cetaceans analyzed.

**Species**	**Collection number**	**Enamel thickness**
**Archaeoceti**		
unnamed Kekenodontidae	OU 22023	180–210 μm
**Platanistoidea**		
unnamed Squalodontidae	OU 22457	350–380 μm
unnamed Squalodontidae	OU 22257	350–380 μm, up to 580 μm at location of enamel ridges
*Otekaikea* sp.	OU 22306	75–85 μm
*Prosqualodon australis*	MPEF-PV 1868	350–380 μm, up to 580 μm at location of enamel ridges
**Delphinoidea**		
unnamed Delphinoidea	OU 22108	100–125 μm
unnamed Delphinoidea	SGO-PV-754	250–300 μm

**Figure 3:**
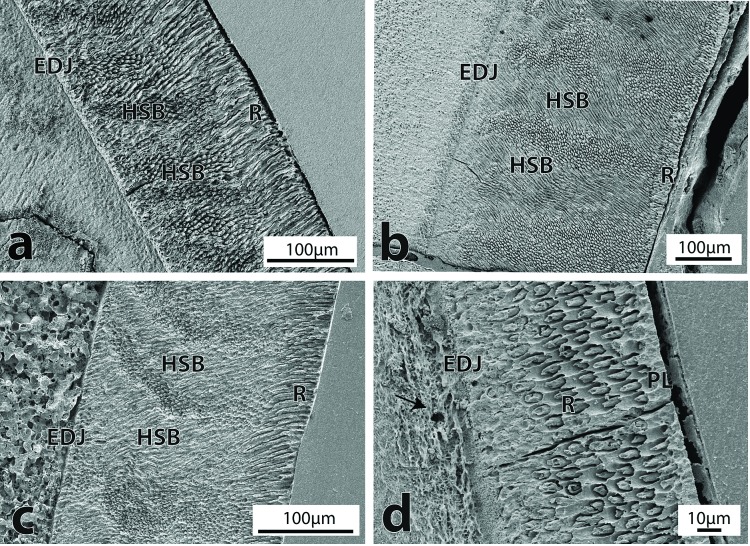
Enamel ultrastructure in Kekenodontidae and Platanistoidea. a) Inner layer of HSB and outer layer of radial enamel in the Kekenodontidae (OU 22023) in longitudinal section, magnification 270X. b) Inner layer of HSB and outer layer of radial enamel in *Prosqualodon* (MPEF-PV 1868) in longitudinal section, magnification 180X. c) Inner layer of HSB and outer layer of radial enamel in the Squalodontidae (OU 22457) in longitudinal section, magnification 230X. d) Radial enamel with thin prismless outer surface in *Otekaikea* sp. (OU 22306) in longitudinal section, magnification 750X. Note tubules near the EDJ (arrow). (EDJ = enamel-dentine junction; HSB = Hunter-Schreger bands; PL = prismless; R = radial).

The enamel *Schmelzmuster* (the spatial distribution of enamel types) for the fossil cetaceans studied, consisted of two different patterns of organization, both of them double-layered. The Platanistoidea and Kekenodontidae shared a Schmelzmuster consisting in an inner layer of Hunter-Schreger bands and an outer layer of radial enamel (Fig. 3A, B and C). This outer layer was about 50 μm thick and the prisms were perpendicular to the enamel-dentine junction (EDJ) and extending towards the outer enamel surface (OES). In the inner HSB layer, a strong decussation of about 90° was seen between bands of prisms. These bands were on average 10–15 prisms thick. Of all the Platanistoidea specimens analyzed here, the only one that lacked this organization was *Otekaikea* sp. (OU 22306). This specimen, together with the two fossil Delphinoidea analyzed, shared a simple organization consisting of an inner layer of radial enamel and a thin outer layer of prismless enamel (Fig. 3D). These three taxa are polydont and homodont, while the other platanistoids and Kekenodontidae are strongly heterodont and with limited polydonty or, for the Kekenodontidae, probably no polydonty.

### Prisms and Interprismatic Matrix (IPM)

For most of the specimens evaluated here, prism cross section in longitudinal and cross sections revealed a basally open sheath, with prisms organized in alternating position consistent with Pattern 3 of Boyde [25]. Closed sheath prisms (Pattern 1 of Boyde) were often common together with open sheath prisms. In *Otekaikea* sp. OU 22306 and in the smaller denticles of the Kekenodontidae OU 22023, closed sheath prisms seemed predominant. The diameter of prisms varied from 3 to 5 μm both in open and closed sheath prisms. Interprismatic matrix (IPM) surrounded the prisms and IPM crystallites were parallel to one another, but at a slight angle to the prism long axes.

### Outer enamel surface (OES) and Enamel-dentinal junction (EDJ)

**Figure 4:**
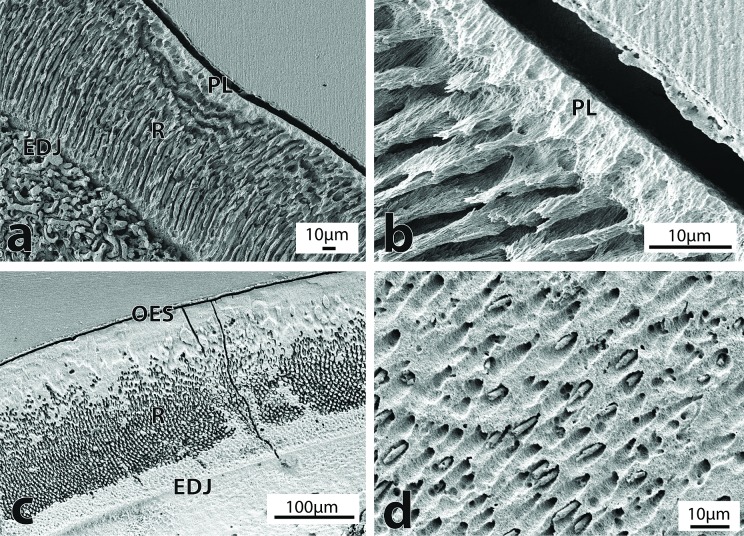
Enamel ultrastructure in Delphinoidea and dentine structure. a) Radial enamel with prismless outer surface in the Delphinoidea (OU 22108) in longitudinal section, magnification 430X. Note heavily altered dentine underneath the EDJ, possibly due to secondary carbonates. b) Detail of the prismless outer layer in the Delphinoidea (OU 22108) in longitudinal section, magnification 2200X. c) Radial enamel with prismless outer surface in the Delphinoidea (SGO-PV-754) in cross section, magnification 210X. Note the diagenetic alteration in the OES and near the EDJ. d) Dentinal surface of *Prosqualodon australis* (MPEF-PV 1868) in longitudinal section, magnification 1200X. Note the mineral crystallization inside dentine tubules. (EDJ = enamel-dentine junction; PL = prismless; OES = outer enamel surface; R = radial).

Two different configurations were found at or near the OES of fossil cetaceans. The fossil Delphinoidea and *Otekaikea* sp. had a thin outer layer of prismless enamel, where hydroxyapatite crystallites were organized parallel to each other and with their long axis directed towards the OES (Fig. 3D, 4A and B). However, the remaining specimens (Squalodontidae, Kekenodontidae and *Prosqualodon*) had an outer layer composed of radial enamel (Fig. 3A, B and C). The prismless layer was very thin (about 10 μm) in the Oligocene *Otekaikea* sp. and the Oligocene Delphinoidea from New Zealand (Fig. 4B), representing about 15–10% of the enamel thickness. The Chilean Delphinoidea had a thicker prismless layer of about 50 μm, but this layer was heavily altered by diagenesis and precise measurements could not be made (Fig. 4C). SEM images showed that this layer was uniformly covered by exogenous mineral material, which made it difficult to observe any crystallite features. In the Squalodontidae, *Prosqualodon* and Kekenodontidae, the radial outer layer was about 50 μm thick, representing from 15 to about 25% of the enamel thickness.

The EDJ was characterized by a sharp and well-defined boundary. A thin layer of prismless enamel a few μm thick was often just above the EDJ. Diagenetic alteration, indicated by exogenous mineralization, was often common at dentine underneath the EDJ zone (Fig. 3C and D, 4A). The subsurface mineralization was not studied in its own right, but surfaces of other teeth and bones sourced from the same strata as the studied teeth have shown secondary carbonates, phosphates, and iron and manganese minerals.

### Tubules and tufts

Tubules and tuft-like structures were seen at the EDJ in some fossil cetaceans both in cross-sectional and longitudinal sections (Fig. 3D). Tubules seen at the EDJ or at the basal enamel measured roughly 0.5 μm or less in diameter. Tuft-like structures were seen at the EDJ and extended to the basal portion of enamel. Tufts were ribbon-like in shape and ran longitudinally towards the OES. Tubules and tuft-like structures were often associated with diagenetic changes and cracks, probably acting as nuclei for later diagenetic changes.

### Dentine

Fossil cetacean dentine was heavily affected by diagenesis, mainly in the form of mineral infillings, mineral growth inside dentine tubules and loss of expected detail of dentine structure (Fig. 4A and D). In less altered regions, fossil dentine was relatively featureless with an uneven surface and irregularly distributed dentinal tubules measuring about 1 μm in diameter.

### Enamel ultrastructure and phylogenetic pattern

**Figure 5:**
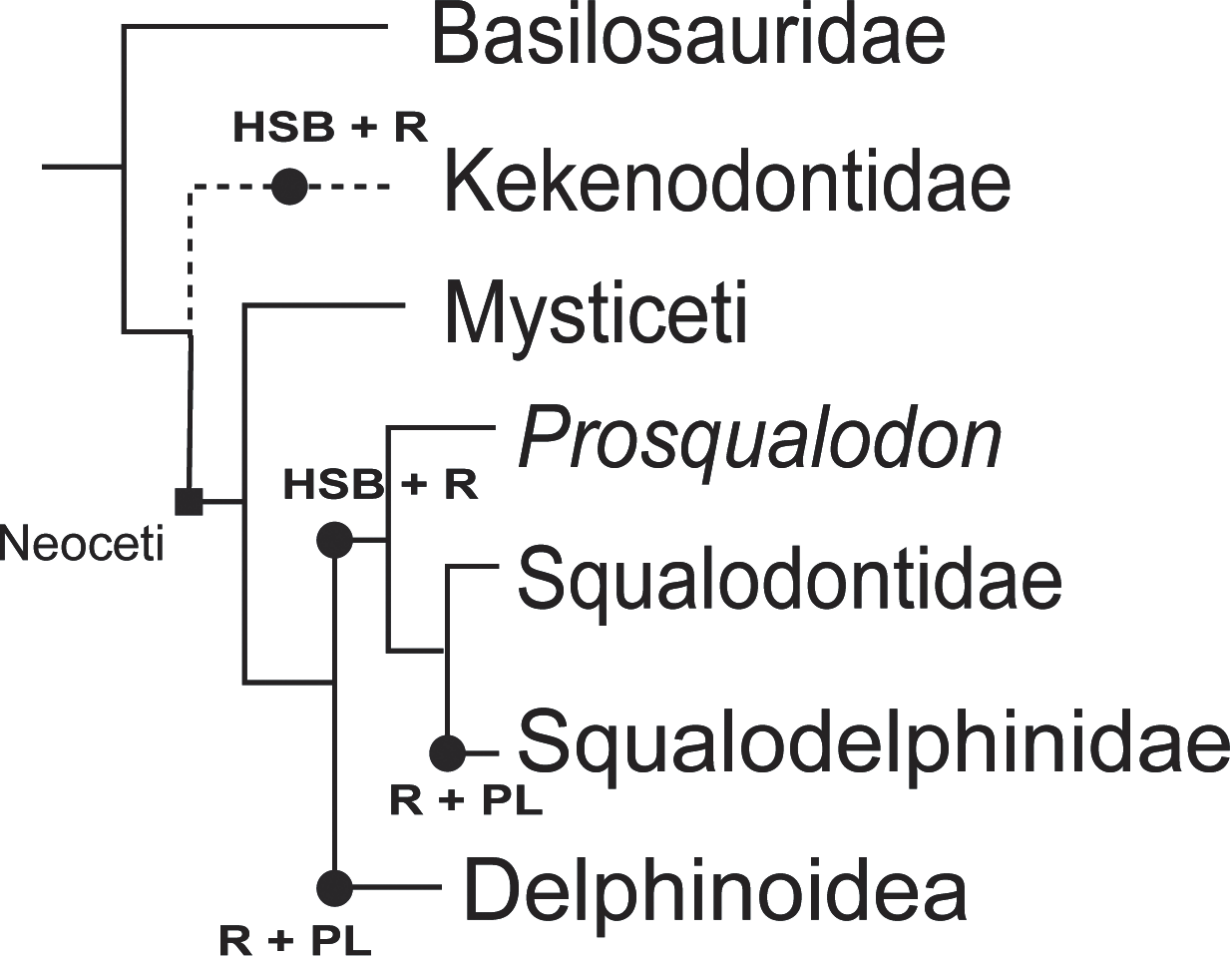
Generalized phylogeny of the fossil cetaceans analyzed in this study, based on Murakami *et al.* 2012 [39], and the *Schmelzmuster* of fossil cetacean enamel (HSB + R indicates Hunter-Schreger bands + radial; R+PL indicates radial + prismless). The phylogenetic position for Kekenodontidae followed Fordyce (2004) [40].

Prismatic enamel was observed in all fossil specimens, with Pattern 3 (open) prisms and Pattern 1 (closed) predominant (patterns from Boyde [25]). At the *Schmelzmuster* level, two main trends were evident when plotted on a simplified cetacean phylogeny (Fig. 5). An inner layer of HSB and an outer layer of radial enamel were observed in Kekenodontidae and in both *Prosqualodon* and Squalodontidae. Conversely, *Otekaikea* sp. (cf. Squalodelphinidae) and both specimens of Delphinoidea had a simpler *Schmelzmuster* composed of radial enamel and an outer layer of prismless enamel.

## Discussion

The dominant prism pattern observed in the fossil cetaceans was an open prism sheath, with prisms horizontally arranged in alternating positions. This arrangement is consistent with the Pattern 3 of Boyde’s classification [25]. Closed prisms were sometimes found near the outer enamel surface (notably in *Otekaikea* sp. and Kekenodontidae), but always less frequent than open sheath prisms. This arrangement, in which open sheath prisms predominate with closed prisms scattered close to the tooth surface, is also a common trend recorded in many terrestrial mammals and archaeocete cetaceans [19,26,27]. The average prism size (maximum diameter) of about 5 μm was similar among all fossil species, independent of differences in tooth size and presumed body sizes, and it was consistent with what was reported for modern humans and other land mammals [28].

At the *Schmelzmuster* level, we observed a complex organization resulting from an inner layer of HSB and an outer layer of radial enamel in the Kekenodontidae (Archaeoceti), and in the Squalodontidae and *Prosqualodon* (Odontoceti). These three extinct taxa are heterodont, with differentiated teeth that include denticulate cheek teeth. HSB have been related to increases in body size and weight during the evolution and diversification of eutherians, and are considered to be a biomechanical reinforcement against enamel cracking in response to increased occlusal loads [17,27,29]. HSB evolved convergently in diverse mammal groups during the Paleocene and Eocene [17]. By the Late Eocene, cetaceans were indeed larger than most contemporary terrestrial mammals [30]. It is possible that macrophagy, HSB, and body size co-evolved in early cetaceans, but testing this hypothesis is a major project in its own right, beyond the scope of this paper. Meanwhile, the occurrence of HSB in some of our study material suggests feeding strategies involving higher occlusal loads in heterodont cetaceans than in homodont forms, whether living or fossil.

HSB were also reported as present in the dentition of basal archaeocetes [18–20] and the extant Ganges River dolphin *Platanista gangetica* [20] and Amazon River dolphin *Inia geoffrensis* [22]. While a thin prismless layer was reported in the OES of the Ganges and Amazon River dolphins [20,22], a moderately thick layer of radial enamel was seen in the OES of all fossil cetaceans with HSB evaluated in this study. This configuration, with prisms perpendicular to the outer enamel surface, was also reported in some basal Eocene archaeocetes [20].

An inner layer of radial enamel and an outer layer of prismless enamel were observed in the fossil *Otekaikea* sp. and Delphinoidea, similar to the structure reported for extant Delphinoidea [21,22]. This organization was the only type of prismatic enamel found in stem mammals as well as in many living marsupials and small-bodied placentals, and it is considered a plesiomorphic *Schmelzmuster* among mammals [27,31]. Large-bodied mammals rarely have radial enamel as the dominant type [31]. Radial enamel is also regarded as more wear-resistant and has been noted in mammalian groups in which opposing tooth surfaces slide over each other [14,31]. Due to the dense packing of crystallites oriented perpendicular to the surface, the outer layer of prismless enamel is considered more resistant to attrition and abrasion than prismatic enamel [27]. An inner layer of radial enamel and outer layer of prismless enamel is the most common *Schmelzmuster* in extant Delphinoidea; such dolphins commonly possess a simplified polydont-homodont dentition in which upper and lower teeth interdigitate to grasp and secure prey but probably do not masticate [22].

In most of the specimens analyzed, dentine was characterized by subparallel tubules embedded in a matrix of intertubular dentine rich in fibers, which may represent collagen fibers that became secondarily mineralized after burial [17,32]. At the EDJ, structures similar to tubules and enamel tufts were often identified. In earlier studies, tubules were also recognized in archaeocetes [20] and recent delphinoids [21,22], possibly related to a low degree of enamel mineralization at the EDJ. Tufts are also regarded as hypomineralized areas of enamel and have been considered as intrinsic crack-like structures that play a major role in damage tolerance and mechanical response due to occlusal loading [33].

The dentine and EDJ were areas that showed clear diagenetic alteration in most of the fossil specimens analyzed. These alterations include recrystallization and presumed-secondary mineral growths (mineral without obvious biogenic microstructure), commonly not observed in the dentine and EDJ of extant cetacean teeth. The composition of these minerals was not determined, but there is independent evidence of secondary carbonates and phosphates in the source rocks. The dentine and EDJ contain dentinal tubules, tuft-like structures, and tubules, all of which are known to act as conduits for diagenetic fluids during fossilization [17]. In the fossils, the enamel layer preserved microstructural details more reliably than dentine, but some fossil specimens also had their enamel layer slightly altered by diagenesis (*e.g.* Chilean Delphinoidea, which is from a sequence known for its secondary phosphate mineralization). These modifications include fusing of enamel crystallites to form a uniform matrix that was more resistant to acid etching, particularly in the OES and close to the EDJ. However, besides these alterations, the overall enamel ultrastructure could be reasonably reconstructed in the Chilean delphinoid.

Major changes in tooth morphology normally result in simplification of enamel microstructure in response to changed biomechanical demands [14]. The simplification in tooth form during the evolution of cetaceans was also reflected in the thickness of the enamel cover. For the fossil specimens analyzed here, enamel was moderately thick in the Squalodontidae, *Prosqualodon* and Chilean Delphinoidea (about 300–380 μm thick), but thinner in the Kekenodontidae, *Otekaikea* sp., and New Zealand Delphinoidea (ranging from 75–200 μm thick). Thus, enamel thickness shows no strong correlation with phylogeny, or tooth-size, or heterodont versus homodont form. In Eocene archaeocetes, enamel thickness was reported as ranging from 400–500 μm, with well-developed HSB and a wide variation of prism cross-section [19,20]. For most mammals, the presence of a thick enamel layer would enhance resistance to contact-induced fracture, and would prolong tooth lifetime in case of progressive wear [34]. Thus, the moderately thin layer of enamel in fossil cetaceans suggests limited utility in feeding and relaxed selection for tooth microstructure in comparison with other eutherians [9].

The occurrence of a double-layered *Schmelzmuster* consisting of an inner layer of HSB and an outer layer of radial enamel in the Kekenodontidae, Squalodontidae and *Prosqualodon*, all of which were heterodont, suggests that Oligocene archaeocetes and early odontocetes inherited the *Schmelzmuster* of more-basal archaeocetes [18–20]. These clades were heterodont and non-polydont (kekenodontids), or heterodont with limited polydonty (Squalodontidae and *Prosqualodon*). Archaeocetes and most stem Oligocene odontocetes probably used their anterior conical teeth to grasp and restrain prey, including fish and other marine vertebrates, while the low-crowned, laterally compressed, denticulate, and ornamented posterior teeth were possibly used to slice and shear [6,35,36]. External surface ornamentation of the enamel seen in many fossil cetaceans could help to grip and seize prey, but the functional and phylogenetic significance of ornament is still unknown. The heavily built, subtriangular, shark-like teeth of squalodontids and other archaic odontocetes suggested to Kellogg (1928) a predaceous feeding habit on bigger fish or even small pelagic mammals [37]. The rostrum in squalodontids and relatives was long and attenuated, suggesting a fast-snap but not necessarily a powerful bite. The short and robust rostrum of *Prosqualodon* would allow a more powerful bite although possibly slower; probably allowing the removal of large chunks of prey [3].

A *Schmelzmuster* consisting of an inner layer of radial enamel and an outer layer of prismless enamel was observed in the squalodelphinid-like dolphin *Otekaikea* sp. and in Delphinoidea, both of which are more-crownward than the Kekenodontidae, Squalodontidae, and *Prosqualodon*. This configuration was also reported as common in extant Delphinida, a clade characterized by a polydont and homodont dentition [22]. *Otekaikea* sp. and the Delphinoidea specimens analyzed here have tooth morphologies consistent with a grasping and piercing action, but not mastication, implying limited food processing [36]. These odontocetes, with many slender and pointed teeth, were probably raptorial predators which used their elongate pincer-like jaws and rostra to secure and pierce grasped prey. Prey items were probably swallowed after limited food processing, as seen in most extant Delphinida [9,38].

The transition from a heterodont and nonpolydont (or limited polydont) dentition in archaeocetes and early odontocetes, to homodont and polydont teeth in more-crownward odontocetes, also marked a simplification in the enamel *Schmelzmuster.* Such trends were likely related to functional changes such as the lack of mastication-related occlusion and less constrained feeding biomechanical demands in the fossil *Otekaikea* sp. and Delphinoidea, as seen in extant Delphinida [22]. These modifications involved particularly the shift in food processing from shear-and-mastication in archaeocetes and early odontocetes, to pierce-and-grasp occlusion in most crownward cetaceans.

It is widely accepted that the evolution of mammalian enamel has been driven by a combination of developmental and geometric constraints, functional influences and phylogenetic history [27]. The phylogenetic significance of some enamel features might be obscured by homoplasy (*e.g.* convergent presence of HSB in different groups), but some evolutionary trends can still be unveiled in clades with common functional adaptations [27]. Further studies considering more specimens of late Oligocene and early Miocene odontocetes would help in elucidating how the transition from HSB to radial enamel has occurred and the role of phylogeny and dental functional biomechanics in the macro and ultrastructure of cetacean enamel.

## Supporting Information

S1 Table List of diagnostic characters used to identify species to family-level. (DOCX)
