# Supplementary material for: Enamel Ultrastructure in Fossil Cetaceans (Cetacea: Archaeoceti and Odontoceti)
Source: PLoS One. 2015 Jan 28;10(1):e0116557. doi: 10.1371/journal.pone.0116557 (PMC4309603; doi:10.1371/journal.pone.0116557)
Supplement: S1 Table — (DOCX) [file pone.0116557.s001.docx]

**Supporting Information**

S1 Table: List of diagnostic characters used to identify species to family-level.

| **Taxa** | **Specimen Collection number** | **Diagnostic characters** |
| --- | --- | --- |
| **Archaeoceti** |  |  |
| Kekenodontidae species unnamed (close to "*Squalodon*" *gambierensis*) | OU 22023 | Tooth from partial skull from Oligocene strata which has: attenuate narrow rostrum with only partly open mesorostral groove closely matching *Kekenodon* sp., OU22294 (of Clementz et al. (2014): Fig. 2 [[1](#_ENREF_1)]); dentition of non-polydont heterodont teeth, with conical anterior and more-complex denticulate posterior teeth. More-derived than Basilosauridae in that periotic lacks an elevated superior process. Closely-matching skull of OU22294 shows other features that place kekenodontids between Basilosauridae and Neoceti: short posterior process not exposed laterally, resulting in amastoid condition shared with Neoceti; more primitive than Neoceti in lacking the ascending process of maxilla (of Odontoceti), and lacking the flat ventral surface of maxilla and infraorbital plate (of Mysticeti). |
| **Platanistoidea** |  |  |
| Squalodontidae unnamed | OU 22457 | Tooth from complete skull from Oligocene strata which has: long, deep and robust rostrum; polydont heterodont teeth; free-standing accessory denticles on and high crowned triangular cheek teeth, somewhat inflated laterally; enamel ornamented with *cristae rugosae* or vertical subparallel ridges; symmetrical cranium with large temporal fossa and *Prosqualodon*-like periotic ("archaic squalodontid" of Fordyce (2011) [[2](#_ENREF_2)], figured by Ichishima and Fordyce (2007): 50 [[3](#_ENREF_3)]) |
|  | OU 22257 | Isolated tooth from Oligocene strata closely matching teeth associated with diagnostic skulls of Squalodontidae (see the skull figured by Ichishima and Fordyce (2007): 49-50 [[3](#_ENREF_3)]), characterised by: high, mesiodistally elongate, slightly inflated crown with a rising inverted-V shape enamelocementum boundary resulting in a figure-8 cross section; enamel finely ornamented with vertical sub-parallel *cristae rugosae* variously associated with basal denticulate cingulum; relatively small accessory denticles free-standing away from mesiodistal keel. |
| *Prosqualodon australis* | MPEF-PV 1868 | Close match to cheek-teeth previously identified from *Prosqualodon australis* (see Lydekker (1894): plate 4 [[4](#_ENREF_4)]) and the closely related *Prosqualodon davidis* (see Flynn (1948): plate 4 [[5](#_ENREF_5)]); from early Miocene Gaiman Formation that produced skulls of *P. australis*. |
| cf. Squalodelphinidae, *Otekaikea* new species | OU 22306 | One of 60 conical-crowned, non-denticulate single-rooted teeth collected with longirostral skull, from Oligo-Miocene boundary strata, of *Otekaikea* (new species, Tanaka and Fordyce submitted), being the sister taxon to *Otekaikea marplesi* (Dickson 1964 [[6](#_ENREF_6)]) sensu Tanaka and Fordyce (2014) [[7](#_ENREF_7)]. |
| **Delphinoidea**  Delphinoidea unnamed | OU 22108 | One of 40 conical robust-crowned, non-denticulate, single- rooted homodont teeth from Oligocene strata collected with a partial skull (inflated braincase), short mandible with a short symphysis, a bulla with a saddle-like profile on the involucrum, and anteroposteriorly compressed cervical vertebrae. |
| Delphinoidea unnamed | SGO-PV-754 | Isolated tooth from Pliocene strata associated with skull remains; conical robust crown, lack of denticles, and single subcylindrical root are consistent with derivation from a moderate-sized delphinid comparable with *Tursiops*, but genus is not currently determinable. |

**S1 References**

1. Clementz MT, Fordyce RE, Peek SL, Fox DL (2014) Ancient marine isoscapes and isotopic evidence of bulk-feeding by Oligocene cetaceans. Palaeogeography, Palaeoclimatology, Palaeoecology 400: 28-40.

2. Fordyce RE (2011) A new late Oligocene archaic squalodontid from New Zealand. Journal of Vertebrate Paleontology, Program and Abstracts 2011: 110.

3. Ichishima H, Fordyce RE (2007) Kujira ga riku o aruite ita koro : kyoryu zetsumetsugo no oja [When the whales roamed on land] [Catalog of an exhibition held at Fukui Kenritsu Kyoryo Hakubutsukan, 2007.]. Katsuyama: Fukui Kenritsu Kyoryo Hakubutsukan. 87 p.

4. Lydekker R (1894) Contributions to a knowledge of the fossil vertebrates of Argentina. Part II. Cetacean skulls from Patagonia. Annales del Museo de La Plata 1893 2: 1-14.

5. Flynn TT (1948) Description of *Prosqualodon davidi* Flynn, a fossil cetacean from Tasmania. Transactions of the Zoological Society of London 26: 153-197.

6. Dickson MR (1964) The skull and other remains of *Prosqualodon marplesi*, a new species of fossil whale. New Zealand Journal of Geology and Geophysics 7: 626-635.

7. Tanaka Y, Fordyce RE (2014) Fossil dolphin *Otekaikea marplesi* (Latest Oligocene, New Zealand) expands the morphological and taxonomic diversity of oligocene cetaceans. PloS One 9: e107972.
